# Performance of the Pentagon Drawing test for the screening of older adults with Alzheimer's dementia

**DOI:** 10.1590/1980-57642018dn12-010008

**Published:** 2018

**Authors:** José Eduardo Martinelli, Juliana Francisca Cecato, Marcos Oliveira Martinelli, Brian Alvarez Ribeiro de Melo, Ivan Aprahamian

**Affiliations:** 1 MS, PhD, Division of Geriatrics and Gerontology, Department of Internal Medicine, Faculty of Medicine of Jundiaí, Jundiaí, SP, Brazil; 2 MD, Division of Geriatrics and Gerontology, Department of Internal Medicine, Faculty of Medicine of Jundiaí, Jundiaí, SP, Brazil; 3 PhD, Division of Geriatrics and Gerontology, Department of Internal Medicine, Faculty of Medicine of Jundiaí, Jundiaí, SP, Brazil; 4 MD, MS, PhD, Division of Geriatrics and Gerontology, Department of Internal Medicine, Faculty of Medicine of Jundiaí, Jundiaí, SP, Brazil

**Keywords:** Alzheimer's disease, cognitive assessment, screening instrument, doença de Alzheimer, avaliação cognitiva, teste de rastreio

## Abstract

The Pentagon Drawing Test (PDT) is a common cognitive screening test.

**Objective:**

The aim of this study was to evaluate performance properties of a specific PDT scoring scale in older adults with Alzheimer's disease (AD) and healthy controls.

**Methods:**

A cross-sectional study of 390 elderly patients, aged 60 years or older with at least two years of education was conducted. All participants completed clinical and neuropsychological evaluations, including the Cambridge Cognitive Examination, the Mini-Mental State Examination (MMSE), and the Clock Drawing Test. All PDT were blindly scored with the scale of Bourke et al.

**Results:**

PDT analyses of the binary score on the MMSE (0 or 1 point) did not discriminate AD from controls (p = 0.839). However, when PDT was analyzed using the Bourke et al. scale, the two groups could be distinguished (p <0.001). PDT was not affected by education, showed sensitivity of 85.5% and specificity of 66.9%, discriminated different clinical stages of dementia, and correlated with the other cognitive tests (p <0.001). A 1-point difference on the Bourke et al. scale was associated with an odds ratio of 3.46 for AD.

**Conclusion:**

PDT can be used as a cognitive screen for suspected cases of dementia, especially AD, irrespective of educational level.

## RESUMO

O teste do desenho do pentágono (PDT) é um teste de rastreio cognitivo simples.

**Objetivo:**

O objetivo deste estudo foi avaliar o desempenho de uma escala específica de pontuação da PDT em idosos com doença de Alzheimer (DA) e controles saudáveis.

**Métodos:**

Estudo transversal, com 390 idosos, com mais de 60 anos de idade, com pelo menos dois anos de escolaridade. Todos os participantes passaram por anamnese clínica e neuropsicológica, incluindo o Cambridge Cognitive Examination (CAMCOG), o Mini-Exame do Estado Mental (MEEM) e o Teste do Desenho do Relógio (TDR). A avaliação do PDT com a escala Bourke et al. foi feita de forma cega.

**Resultados:**

As análises PDT do escore binário do MEEM (0 e 1 ponto) não discriminaram DA dos controles (p = 0,839). Contudo, quando PDT foi avaliada pela escala Bourke et al., verificou-se diferenças estatisticamente significativa (p <0,001). A PDT não sofreu interferência da escolaridade, apresentando sensibilidade de 85,5% e especificidade de 66,9% para discriminar os diferentes estágios clínicos da demência. A escala também mostrou correlação com os testes cognitivos aplicados (p <0,001). Uma diferença de um ponto na escala Bourke et al. foi associada com OR (odds ratio) de 3,46 para DA.

**Conclusão:**

PDT pode ser utilizada como rastreio cognitivo para casos suspeitos de demência, especialmente DA, independentemente.

Praxis refers to a complex cognitive function, in which the motor system is used to execute complex learned actions and movements, such as the copying of drawings.^1^ Praxis evaluation is an important stage in the cognitive evaluation of older adults because apraxia may suggest the presence of neurodegenerative disease, such as Alzheimer's disease (AD),^2^^,^ 3 vascular dementia^4^^,^ 5 or Parkinson's disease,^6^^,^ 7 among others. Apraxia is included as one of the cognitive domains evaluated in the diagnostic criteria of major neurocognitive disorder according to the DSM-5.^8^ Praxis functions depend mainly on the parietal lobe, which is specialized for visuospatial information, visual attention, body position, whole object perception relative to self, among other functions, but it is well accepted that apraxia can be the result of a more global cognitive dysfunction.^9^

The Mini-Mental State Exam (MMSE) is one of the most used screening tests to evaluate cognitive impairment in older adults.^10^ It is a simple 30-point screening tool for evaluating mental status, assessing recent memory, orientation, attention, language and praxis. The sub-tests of the MMSE score 6 points for memory, 10 for orientation, 5 for attention, 8 for language and only 1 for praxis, which is evaluated by drawing two intersecting pentagons, in which the interconnected area should be shaped like a rhombus.^11^^,^ 12 The interpretation of the pentagon drawing test (PDT) is usually binary, with 1 point for the correct figure.

**Figure 1:**
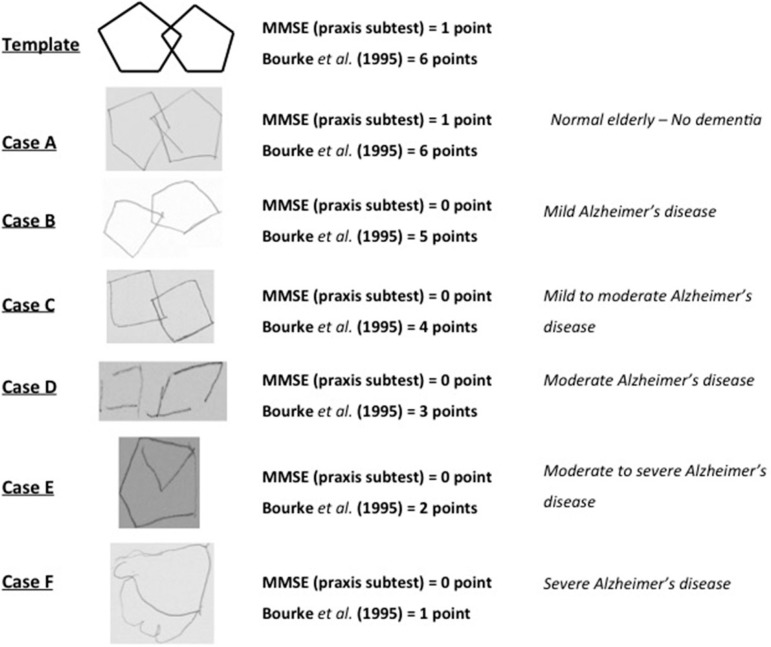
Template and clinical examples of the Pentagon Drawing Test in preserved older adults and Alzheimer's dementia.

The PDT can be used for the evaluation of praxis function, but also may predict a global cognitive dysfunction. There are several ways of interpreting the test, besides the binary method used in the MMSE.^1^^,^ 12 Bourke et al. (1995) described an easy scoring scale for the PDT with a total of 6 points.^9^ One point is given for lines drawn or attempt to draw a figure, 2 for drawing a figure, 3 for two figures not overlapping; 4 for two overlapping figures, 5 for an overlapping connection and one figure being a pentagon, and 6 for the correct copy. The cut-off score established by the validation study was >5 points.

The primary aim of this study was to compare the performance on the PDT according to the Bourke et al. scoring scale between healthy older adults and patients with AD. Additionally, we evaluated whether the PDT is negatively influenced by educational level. We hypothesize that the PDT can discriminate between these groups if analyzed by a more detailed scale than a simple binary analysis.

## METHODS

### Participants

This cross-sectional study was based on a cognitive cohort of older adults. In the present study, a convenience sample of 390 older adults was evaluated at the Geriatrics Outpatient Center of the Faculty of Medicine of Jundiaí between March 2010 and May 2015. The city of Jundiaí is located in the Southeastern region of Brazil in the state of São Paulo. It has a population of 397,965 and is ranked the fourth city for human development and eighth in terms of economy in the state.

Individuals of both sexes, aged 60 years or older and with at least two years of education, underwent a broad clinical and neuropsychological evaluation. After the initial assessment and a diagnostic consensus meeting, all participants were classified into two diagnostic groups: probable AD (n = 266) and normal control (NC, n = 124). The NC group comprised older adults that were followed for clinical comorbidities other than neurological or psychiatric disorders, and did not present any consistent subjective memory or behavior complaints during the clinical interview. Both groups had similar monthly income of around 797.58 US dollars (equivalent to three minimum wages in Brazil). The study is in accordance with the ethical standards of the Committee on Human Experimentation of the Institution as well as compliant with the Helsinki Declaration. All patients and their legal guardians agreed to participate by signing an informed consent protocol approved by the local Ethics Committee (protocol number: 54/11).

### Inclusion and exclusion criteria

The NC group comprised older adults that did not present any cognitive or behavior complaints, or activities of daily living impairments according to patients and their family members (Pfeffer Functional Activities Questionnaire^13^ <5 points). The controls had normal performance on the neuropsychological tests (i.e. above the cutoff for cognitive impairment). Inclusion criteria for probable AD met the National Institute of Aging and the Alzheimer's Association recommendations^14^ and the DSM-IV for dementia.^15^ Patients with mild (n = 42), moderate (n = 131) and severe (n = 93) probable AD were included according to the Clinical Dementia Rating score of 1, 2, and 3, respectively. Participants were not included if they presented with depressive symptoms or clinical depression (Geriatric Depression Scale-15 items^16^ >5 points), history of stroke, any limb plegia or paresis, significant tremor, functional impairment in hands, severe visual or auditory impairment, presence of any kind of substance abuse, non-Alzheimer's dementia, and refused to complete any of the tests or sign the informed consent form.

### Procedures

First, all participants were submitted to a detailed in-person clinical interview, and a complete clinical, neurological and psychiatric evaluation. The Mini-Mental State Exam (MMSE),^11^ the Clock Drawing Test (CDT) scored according to Mendez' scale,^17^ the Pfeffer Functional Activities Questionnaire,^13^ and the Geriatric Depression Scale (GDS)^16^ were performed at this time. Also, an interview was conducted separately with a family member. Included patients underwent magnetic resonance of the brain and complete laboratory exams. After a mean of 30 days, all participants were submitted to neuropsychological evaluation, including the Cambridge Cognitive Examination (CAMCOG).^18^ The neuropsychologist who applied the cognitive tests during the first interview and the neuropsychological evaluation did not participate in the diagnostic process and was blind to clinical information regarding the patient. All PDTs were blindly scored for diagnosis according to the scale described by Bourke et al.^9^ (see clinical examples of the scale in Figure 1.

### Statistical analysis

All data were analyzed using the SPSS program version 19.0. Non-parametric distribution was tested by the Kolmogorov-Smirnov test (MMSE mean 21.59 ± 6.14 *p* <0.001; CAMCOG mean 70.54 ± 20.80 *p* = 0.003; PDT by Bourke et al. mean 4.77 ± 1.56 *p* <0.001). The variables were expressed as frequencies, percentages, means and standard deviations. Categorical variables were analyzed by the Chi-squared test, and continuous variables by the Mann-Whitney test or Kruskal-Wallis test (3 or more groups). The two groups (AD and NC), classified according to PDT performance, were tested for correlation with age, education, the MMSE, CAMCOG, and CDT. Logistic regression was used to test the association between PDT score and AD. Receiver operating characteristic (ROC) analyses were performed to assess sensitivity, specificity, and areas under the curve (AUCs) of the PDT, the MMSE, and the CAMOCG. Level of statistical significance was set at 0.05 in two-tailed tests.

## RESULTS

A total of 390 individuals were evaluated. Mean age was 77.78 ± 7.96 years and 71% were women. Regarding education, 57.4% had 2-4 years, 18.5% 5-8 years, and 24.1% had >8 years (Table 1). The AD group corresponded to 68.2% of the sample. This group was older, had a higher proportion of women, and was less educated than the control group (Table 1). The NC group consisted of 124 individuals. Only gender did not show a statistical difference (*p* = 0.146) (Table 1). The neuropsychological assessment using PDT scores for the MMSE and the Bourke et al. scale are summarized in Table 1. There was no difference between the groups according to scores on the PDT from the MMSE (*p* = 0.839). However, a significant difference was observed between the groups for the PDT scored by the Bourke et al. scale (*p* <0.001), and the NC group had a higher correct drawing percentage (85.5%) when compared with the AD group (66.9% incorrect drawing). Additionally, PDT scores differed significantly between AD and NC groups across all educational levels (*p* <0.001) (Table 2).

**Table 1:** Characteristics of the sample regarding age, sex and education.

		NC	AD	*p*
Age, y	mean±SD, range	74.85 ± 6.79 (60-103)	79.30 ± 6.79 (61-98)	0.001**
Gender (%)	Female	83 (66.9%)	197 (74.1%)	0.146*
	Male	41 (33.1%)	69 (25.9%)	
	MMSE (mean±SD)	28.50 ± 1.92	18.44 ± 4.48	
Education	2 to 4 years	52 (41.9%)	172 (64.7%)	0.001**
	5 to 8 years	30 (24.2%)	42 (15.8%)	
	>8 years	42 (33.9%)	52 (19.5%)	

y: years; NC: normal control group; AD: Alzheimer's disease group; SD: standard deviation; MMSE: Mini-Mental State Examination.

* Chi-square test;

** Mann-Whitney test.

**Table 2:** Analysis of the PDT by MMSE binary method and Bourke et al. according to dementia stage.

Interpretation	Mild CDR 1.0 N (%)	Moderate CDR 2.0 N (%)	Severe CDR 3.0 N (%)	p*
MMSE correct drawing ("1" point)	29 (69%)	40 (30.5%)	16 (17.2%)	<0.001
MMSE incorrect drawing ("0" points)	13 (31%)	91 (69.5%)	77 (82.8%)	
Bourke et al. - 1 point	0	5 (3.8%)	11 (11.8%)	<0.001
Bourke et al. - 2 points	1 (2.4%)	14 (10.7%)	22 (23.7%)	
Bourke et al. - 3 points	1 (2.4%)	12 (9.2%)	12 (12.9%)	
Bourke et al. - 4 points	3 (7.1%)	25 (19.1%)	16 (17.2%)	
Bourke et al. - 5 points	7 (16.7%)	34 (26%)	15 (16.1%)	
Bourke et al. - 6 points	30 (71.4%)	41 (31.3%)	17 (18.3%)	

PDT: Pentagon Drawing Test; MMSE: Mini-Mental State Examination; N: number of individuals; NC: normal control group; AD: Alzheimer's disease group.

* Kruskal-Wallis test.

PDT performance was compared for the clinical stage of dementia, i.e. mild, moderate and severe. Both the scores on the MMSE and the Bourke et al. scale were different in mild, moderate and severe stages of dementia, as shown in Table 2. In the AD group, there was a moderate positive correlation between the Bourke et al. scale and the MMSE total score (*r* = 0.57, *p* <0.001), the CAMCOG (*r* = 0.56, *p* <0.001) and the CDT scored by the Mendez scale (*r* = 0.52, *p* <0.001), as shown in Table 3. A weak negative correlation was observed with age (*r* = -0.27, *p* = 0.003). In the NC group, a moderate correlation was found between the Bourke et al. scale and the CDT scored by Mendez (*r* = 0.52, *p* <0.001). Weak correlations were also found between the Bourke et al. scale and the MMSE total score (*r* = 0.39, *p* <0.001) and the CAMCOG (*r* = 0.36, *p* <0.001).

**Table 3:** Correlation between the PDT by Bourke et al. and age, education, MMSE total score, CAMCOG, and CDT.

		Age	Education	MMSE	CAMCOG	CDT Mendez
PDT NC group	*R*	-.267	0.27*	.393	.363	.524
	*p* (2-tailed)	0.003	0.002	<0.001	<0.001	<0.001
	N	124	124	124	124	124
PDT AD group	*R*	-.005	0.34*	.568	.557	.596
	*p* (2-tailed)	0.457	<0.001	<0.001	<0.001	<0.001
	N	266	266	266	266	266

PDT: Pentagon Drawing Test; MMSE: Mini-Mental State Examination; CAMCOG: Cambridge Cognitive Test; CDT Mendez: Clock Drawing Test score according to Mendez scoring scale; NC: normal control group; AD: Alzheimer's disease group; N: number of individuals; *r*: Pearson correlation coefficient.

* Correlation calculated by Spearman.

The PDT scored by Bourke et al. showed lower accuracy when compared to the MMSE and the CAMCOG for discriminating between AD and controls (Table 4). The best PDT cut-off score was >4 in our sample according to the Youden index analysis of the ROC curves. No difference was found between the standard MMSE and the modified version of the MMSE substituting the traditional pentagon drawing score of 0 or 1 point by the Bourke et al. scoring scale based on a 35-point scale. The MMSE and the CAMCOG showed similar accuracy and both had larger areas under the ROC curves (Table 4). A logistic regression model was used to test the association between PDT scoring and AD diagnosis, as well as the influence of age and education, which differed significantly between AD and NC groups (Table 5). From Table 5A, we note that the interaction between the independent variables PDT and education was not significant, indicating that the effect of the PDT on the probability of AD diagnosis was the same across all 3 levels of education. In Table 5B, we consider a logistic model with only the main effects of the independent variables age, PDT and education. The probability of a positive AD diagnosis is not affected by educational level. Notwithstanding, age affected the diagnosis positively (the higher the age, the greater the probability of AD) and the PDT negatively, which means that the higher the PDT score according to Bourke et al., the lower the probability of the patient having AD (odds ratio of 0.29 for higher scoring). For example, if a patient scores one point less on the PDT than another patient, then this individual has a 3.45 (OR should be 1/0.29) greater chance of having AD, if both patients have the same age and level of education.

**Table 4:** Receiver operating characteristic curve areas, cut-offs, sensitivity, and specificity for the PDT, MMSE, MMSE plus the PDT and the CAMCOG between AD and NC groups.

	AUC*	Cut-off**	Sensitivity	Specificity	95% CI
PDT	0.784	≥ 5	95.2%	45.9%	0.738-0.828
MMSE	0.975	≥ 25	96%	90.2%	0.960-0.991
MMSE + PDT^#^	0.973	≥ 30	95.2%	90.2%	0.957-0.989
CAMCOG	0.970	≥ 78	97.6%	88.7%	0.954-0.986

PDT: Pentagon Drawing Test; MMSE: Mini-Mental State Examination; CAMCOG: Cambridge Cognitive Test; NC: normal control; AD: Alzheimer's disease;

* *p* <0.001 for PDT vs MMSE and PDT vs CAMCOG;

** cut-off for best balance between sensitivity and specificity for this sample;

# The traditional score of 0 or 1 point for the pentagon drawing was substituted by Bourke et al. scoring scale with 0 to 6 points. Thus, MMSE total score was 35 points. 95% CI : Asymptotic 95% Confidence Interval.

**Table 5:** Logistic regression analysis of association between PDT scoring and AD.

**A. Estimates of logistic regression model with AD as the dependent variable and age, PDT and interaction between PDT and education as independent variables.**					
**Effect**	**Estimate**	**SE**	**z value**	***p***	**OR**
Intercept	2.91	1.66	1.75	0.08	
Age	0.06	0.02	3.43	0.00	-
PDT	-1.21	0.19	-6.32	0.00	-
PDT+Education group 1* ^#^	-0.09	0.06	-1.44	0.15	-
PDT+Education group 2* ^¶^	0.01	0.05	0.15	0.88	-
**B. Estimates of logistic regression model with diagnosis of AD as the dependent variable and age, PDT and education as independent variables.**					
**Effect**	**Estimate**	**SE**	**z value**	***p***	**OR**
Intercept	3.04	1.67	1.82	0.07	-
Age	0.06	0.02	3.45	0.00	1.06
PDT	-1.24	0.19	-6.52	0.00	0.29
Education group 1	-0.51	0.34	-1.49	0.14	0.60
Education group 2 ^¶^	0.09	0.30	0.29	0.77	1.09

PDT: Pentagon Drawing Test; AD: Alzheimer's disease; SE: standard error; OR: odds ratio;

# group with 5-8 years of education;

¶ group with >8 years of education;

* interaction analysis between PDT and education.

## DISCUSSION

Quick and simple cognitive screening should be the initial evaluation of older patients with suspected cognitive impairment. Currently, the MMSE or the Montreal Cognitive Assessment (MoCA) are among the most commonly used tests recommended for this purpose in clinical practice. Some sub-items of these tests may contribute variably to their total score.^19^^,^ 20 In the present study, we evaluated the praxis, visuospatial and global cognitive function with the PDT according to the Bourke et al. scoring scale.^9^ The PDT discriminated healthy controls from patients with AD, and identified different clinical stages of their illness. Additionally, the PDT interpreted by Bourke et al. correlated significantly with the MMSE, the CAMCOG, and with another visuoconstructional test, i.e. the CDT. As a screening test, the use of the PDT showed sensitivity of over 80% for differentiating AD from controls. Also, the PDT was not affected by educational level, which is important in developing countries and particularly helpful to primary care providers in avoiding multiple-scoring according to different educational backgrounds.

Constructional praxis declines with age and can become impaired by several neurodegenerative diseases.^21^ Despite its importance, usual tasks to evaluate this function, such as the copying of drawings or drawing three-dimensional shapes, are not included in several screening tests. One possible explanation for this is the fact that very few patients complain of constructional apraxia. Most patients and their families usually seek a clinician for concerns about memory or other behavior disturbances. Previously, the use of the pentagon drawing test alone was more commonly associated with the motor evaluation of Parkinson's disease.^22^ However, the use of the PDT can help evaluate global cognitive function besides praxis.^23^

Our study compared the diagnostic performance of the PDT for differentiating AD from normal controls, according to a specific scoring scale for this test. Previously, only nine cohort or case-control studies have evaluated global cognition or visuoconstructional ability through the performance on the PDT.^6^^,^ 13 ^,^ 20 ^,^ 24 ^-^ 29 Two of these studies did not find that the PDT was useful for this purpose.^29^ The first of these showed that the interpretation of the PDT could be negatively influenced by very low educational background.^28^ In our study, almost 57% of the whole sample had less than 5 years of education. Despite this, the PDT significantly discriminated between two different cognitive groups. The second negative study conducted by Mai et al. compared the MMSE PDT to the executive subtests of the MoCA (i.e. Trails B, the copy of a three-dimensional cube, and the CDT) in patients with stroke or transient ischemic attack.^29^ The MoCA subtests were superior to the binary interpretation of the MMSE PDT. The use of detailed scales in the interpretation of the PDT could increase the test's accuracy when compared to the binary method according to previous studies, but warrants further research.^6^^,^ 23 ^-^ 27

Previously, the PDT interpreted by scoring scales other than a binary method helped discriminate between patients with dementia of Lewy body and AD.^6^^,^ 24 ^,^ 25 ^,^ 27 The performance on the PDT of patients with Lewy body dementia was lower and dissociable from their global cognitive impairment when compared with older adults with AD. Also, the test was more sensitive for detecting visuospatial impairments. In one of these studies, the PDT was significantly correlated with MMSE scores.^6^ Our study also showed a positive moderate correlation between the PDT and other cognitive tests (i.e. the MMSE, the CAMOCG, and the CDT). The original study conducted by Bourke et al. also showed a moderate correlation between the PDT and these tests.^9^ Even simple instruments, with an emphasis on global cognitive screening, but which are intended to identify specific cognitive deficits (i.e. the PDT or the CDT and visuospatial or praxis dysfunction), may identify only a small portion of these patients.^30^^,^ 31

In our study, no negative educational effect was observed on the PDT test. This is of the utmost importance in Brazil. Several traditional cognitive screening instruments are influenced by educational background, such as the MMSE and the MoCA.^32^ Even, fast and simple graphical tests such as the Clock Drawing Test, conceptually similar to the PDT, can have its result biased by low education.^33^ Cognitive screening tests must be user friendly and easy to use in order to be considered clinically relevant to public health providers.

Some limitations must be addressed. Our study involved community-dwelling elderly subjects that sought medical attention for general geriatric care. Thus, our results should not be extrapolated to clinical samples from memory clinics that could have a distinct cognitive and behavior profile. Second, our sample was not matched for age and education. However, our research team took care to ensure the veracity of inclusion and exclusion criteria, as well as participant's charts and files of all data in the study.

In conclusion, the PDT can be used as a cognitive screening test to identify AD patients. The instrument showed good correlation with the MMSE, the CDT, and the CAMCOG, and was not affected by educational level. The use of the PDT combined with other brief cognitive screening tests should be explored in future studies.

## References

[1] Strub RL, Black FW (1999). The mental status examination in neurology.

[2] Aguirre-Acevedo DC, Lopera F, Henao E, Tirado V, Muñoz C, Giraldo M (2016). Cognitive Decline in a Colombian Kindred with Autosomal Dominant Alzheimer Disease: A Retrospective Cohort Study. JAMA Neurol.

[3] Ward LS, Cecato JF, Aprahamian I, Martinelli JE (2015). Assessment for apraxia in Mild Cognitive Impairment and Alzheimer's disease. Dement Neuropsychol.

[4] Jodzio K (1995). Neuropsychological description of memory impairment following cortical and subcortical brain injuries. Psychiatr Pol.

[5] Thajeb P, Thajeb T, Dai D (2007). Cross-cultural studies using a modified mini mental test for healthy subjects and patients with various forms of vascular dementia. J Clin Neurosci.

[6] Cormack F, Aarsland D, Ballard C, Tovée MJ (2004). Pentagon drawing and neuropsychological performance in Dementia with Lewy Bodies, Alzheimer's disease, Parkinson's disease and Parkinson's disease with dementia. Int J Geriatr Psychiatry.

[7] Martinelli JE, Santos LMP, Aramaki FO, Montiel JM, Cecato JF (2014). Sugestões de tratamento na doença de Parkinson: intervenções psicomotoras com vídeo game. Geriatr Gerontol Aging.

[8] American Psychiatric Association (2014). Diagnostic and statistical manual of mental disorders DSM-V.

[9] Bourke J, Castleden CM, Stephen R, Dennis M (1995). A comparison of clock and pentagon drawing in Alzheimer's disease. Int J Geriatr Psychiatry.

[10] Moraes C, Pinto JA, Lopes MA, Litvoc J, Bottino CM (2010). Impact of sociodemographic and health variables on mini-mental state examination in a community-based sample o folder people. Eur Arch Psychiatry Clin Neurosci.

[11] Folstein MF, Folstein SE, McHugh PR (1974). Mini-Mental State: a practical method for grading the cognitive state of patients for the clinician. J Psychiatr Res.

[12] Fountoulakis K, Siamouli M, Oral T (2011). The standardized copy of pentagons test. Ann Gen Psychiatry.

[13] Pfeffer RI, Kurosaki TT, Harrah CH, Chance JM, Filos S (1982). Measurement of functional activities in older adults in the community. J Gerontol.

[14] McKhann GM, Knopman DS, Chertkow H, Hyman BT, Jack CR, Kawas CH (2011). The diagnosis of dementia due to Alzheimer's disease: recommendations from the National Institute on Aging-Alzheimer's Association workgroups on diagnostic guidelines for Alzheimer's disease. Alzheimers Dement.

[15] American Psychiatric Association (1994). Diagnostic and statistical manual of mental disorders DSM-IV.

[16] Yesavage JA, Brink TL, Rose TL, Lum O, Huang V, Adey M, Leirer VO (1983). Development and validation of a geriatric depression screening scale: a preliminary report. J Psychiatr Res.

[17] Mendez MF, Ala T, Underwood K (1992). Development of scoring criteria for the clock drawing task in Alzheimer's disease. J Am Geriatr Soc.

[18] Roth M, Tym E, Mountjoy CQ, Huppert FA, Hendrie H, Verma S, Goddard R (1986). CAMDEX. A standardised instrument for the diagnosis of mental disorder in the elderly with special reference to the early detection of dementia. Br J Psychiatry.

[19] Cecato JF, Martinelli JE, Izbicki R, Yassuda MS, Aprahamian I (2016). A subtest analysis of the Montreal cognitive assessment (MoCA): which subtests can best discriminate between healthy controls, mild cognitive impairment and Alzheimer's disease?. Int Psychogeriatr.

[20] Diniz BS, Yassuda MS, Nunes PV, Radanovic M, Forlenza OV (2007). Minimental State Examination performance in mild cognitive impairment subtypes. Int Psychogeriatr.

[21] Buchman AS, Wilson RS, Leurgans SE, Bennett DA, Barnes LL (2015). Change in Motor Function and Adverse Health Outcomes in Older African Americans. Exp Gerontol.

[22] Agostino R, Berardelli A, Formica A, Stocchi F, Accornero N, Manfredi M (1994). Analysis of repetitive and nonrepetitive sequential arm movements in patients with Parkinson's disease. Mov Disord.

[23] Helmes E (2013). Cognitive screening of older adults: the utility of pentagon drawing. Int Psychogeriatr.

[24] Caffarra P, Gardini S, Dieci F, Copelli S, Maset L, Concari L (2013). The qualitative scoring MMSE pentagon test (QSPT): a new method for differentiating dementia with Lewy Body from Alzheimer's disease. Behav Neurol.

[25] Mitolo M, Salmon DP, Gardini S, Galasko D, Grossi E, Caffarra P (2015). The new Qualitative Scoring MMSE Pentagon Test (QSPT) as a valid screening tool between autopsy-confirmed dementia with Lewy bodies and Alzheimer's disease. J Alzheimers Dis.

[26] Kaul S, Elble RJ (2014). Impaired pentagon drawing is an early predictor of cognitive decline in Parkinson's disease. Mov Disord.

[27] Ota K, Murayama N, Kasanuki K, Kondo D, Fujishiro H, Arai H (2015). Visuoperceptual assessments for differentiating dementia with Lewy bodies and Alzheimer's disease: illusory contours and other neuropsychological examinations. Arch Clin Neuropsychol.

[28] Raina SK, Maria A, Chander V, Raina S (2015). Intersecting pentagons as surrogate for identifying the use of mini mental state examination in assessment of dementia in a largely illiterate population. J Postgrad Med.

[29] Mai LM, Sposato LA, Rothwell PM, Hachinski V, Pendlebury ST (2016). A comparison between the MoCA and the MMSE visuoexecutive subtests in detecting abnormalities in TIA/stroke patients. Int J Stroke.

[30] Rinaldi MC, Piras F, Pizzamiglio L (2010). Lack of awareness for spatial and verbal constructive apraxia. Neuropsychologia.

[31] Canzano L, Scandola M, Gobbetto V, Moretto G, D'Imperio D, Moro V (2016). The Representation of Objects in Apraxia: From Action Execution to Error Awareness. Front Hum Neurosci.

[32] Yassuda MS, da Silva HS, Lima-Silva TB, Cachioni M, Falcão DVDS, Lopes A (2017). Normative data for the Brief Cognitive Screening Battery stratified by age and education. Dement Neuropsychol.

[33] Aprahamian I, Martinelli JE, Neri AL, Yassuda MS (2010). Clock Drawing Test accuracy compared to standard screening tests in Alzheimer's disease: results from a study in a sample of Brazilian elderly with heterogeneous educational background. Int Psychogeriatr.

